# Home‐based preparation approaches altered the availability of health beneficial components from carrot and blueberry

**DOI:** 10.1002/fsn3.462

**Published:** 2017-03-18

**Authors:** Boyan Gao, Lu Yu, Jie Liu, Thomas T. Y. Wang, Yinghua Luo, Liangli (Lucy) Yu, Huijuan Zhang, Lingxiao Gong, Jing Wang

**Affiliations:** ^1^ Beijing Advanced Innovation Center for Food Nutrition and Human HealthBeijing Technology & Business University (BTBU)BeijingChina; ^2^ Department of Nutrition and Food ScienceUniversity of MarylandCollege ParkMDUSA; ^3^ Beijing Higher Institution Engineering Research Center of Food Additives and Ingredients Beijing Technology & Business University (BTBU)BeijingChina; ^4^ DietGenomics and Immunology LaboratoryUSDA‐ARSBeltsvilleMD

**Keywords:** blueberry, carrot, high performance liquid chromatography (HPLC), radical scavenging, total phenolic contents (TPC)

## Abstract

This study investigated the effects of different home food preparation methods on availability of total phenolic contents (TPC) and radical scavenging components, as well as the selected health beneficial compounds from fresh blueberry and carrot. High performance liquid chromatography (HPLC) analysis revealed that ground carrots using blenders released significantly greater amount of β‐carotene than their chopped counterpart, and blueberry samples prepared with different commercial blenders/grinders released different levels of cyaniding‐3‐*O*‐glucoside and malvidin‐3‐*O*‐glucoside. Furthermore, microwaving was able to significantly alter the releasable amounts of cyaniding‐3‐*O*‐glucoside and malvidin‐3‐*O*‐glucoside from blueberries. In addition, carrots and blueberries processed using different blenders and chopping with or without microwaving differed in their available levels of TPC, and radical scavenging components against DPPH ^•^, oxygen radicals and HO ^•^, as well as their potential anti‐inflammatory activities. Taking together, these results indicated that home food preparation approaches may alter the availability of health components from carrots and blueberries. The results also suggested that the influence may depend on the fruit and vegetable type, but not the price of blenders/grinders.

## 1 Introduction

Fruits and vegetables are recognized for their health‐promoting potentials due to their high concentrations of health beneficial components, including phenolic acids, flavonoids, anthocyanins, and other bioactivity components (Cao, Booth, Sadowski, & Prior, 1998; John, Ziebland, Yudkin, Roe, & Neil, 2002; Samman et al., 2003). Carrot (*Daucus carota subsp. sativus*) is one of the most popular and widely consumed vegetables all over the world. The Food and Agriculture Organization reported that the annual human carrot consumption is about 37 million tons, and this number keeps on increasing (FAOSTAT, 2013). Carrot is rich in edible fiber, carotenoids, and other health beneficial compounds (Azam, Khan, Mahmood, & Hameed, 2013; Knockaert et al., 2013). Its carotenoids including β‐carotene are important for vision health, and could not be synthesized by human body and have to be obtained through diet (Albanes et al., 1995; Bendich & Shapiro, 1986; Knockaert et al., 2013).

Blueberry (*Vaccinium corymbosum*) is one of the most popular and important berry fruits globally. It contains significant amount of phenols, including phenolic acids, flavonoids, anthocyanins and procyanidins, which have been reported for many health benefits to human beings, such as improving cardiovascular health (Riso et al., 2013), brain health and insulin sensitivity (Whyte & Williams, 2015), as well as strong antioxidant activities (Gu et al., 2002; Kang, Thakali, Jensen, & Wu, 2015).

Recent studies indicated that different food preparation methods could significantly change the chemical profiles and bioactivities including antioxidant activity of foods. For example, green tea powders with different particle size showed different antioxidant activities (Zaiter, Becker, Karam, & Dicko, 2016). Also noted was that different treatment could influence the antioxidant properties and phenolic compounds in rice bran and husk (Wanyo, Meeso, & Siriamornpun, 2014). In addition, a previous study suggested that coriander powder in different particle sizes showed different antioxidant activities (Barnwal, Singh, Sharma, Choudhary, & Saxena, 2015). A previous study from our group also observed that the reduction in particle size could effectively increase the availability of wheat antioxidant abilities (Cheng et al., 2006). Therefore, this study evaluated whether and how different home‐based food preparation methods, including blending, chopping with/without microwaving, might release different levels of beneficial bioactives from carrot and blueberry food models. The results of this study may be used to improve human health through improving home‐based food preparations.

## 2 Materials and methods

### 2.1 Materials and chemicals

Carrot and blueberry samples were purchased from a local supermarket. Five home‐use blenders, including Nutribullet 600, Nutribullet Pro 900, Nutribullet RX, Vitamix 5200 and Oster Versa 1400 were gifted from Capital Brans, LLC., Los Angeles, California.

ABTS chromophore, diammonium salt, iron (III) chloride, fluorescein (FL), 6‐hydroxy‐2,5,7,8‐tetramethylchroman‐2‐carboxylic acid (Trolox), 2,2‐diphenyl‐1‐poicrylhydrazyl (DPPH), tretrazolium blue (NBT), xanthine oxidase (XOD), hypoxanthine (HPX), manganese dioxide, hydrogen peroxide, Folin‐Ciocalteu (FC) reagent, sodium carbonate, gallic acid, β‐carotene, malvidin‐3‐O‐glucoside and cyanidin‐3‐*O*‐glucoside were analytical grade and purchased from Sigma‐Aldrich (St. Louis, MO). HPLC grade water, methanol, acetonitrile, and formic acid were purchased from Fisher Scientific (Waltham, MA).

### 2.2 Sample preparation

Carrots were cut into one‐inch length, accurately weighted and blended with pure water (1:2, w/v) for 20 s using the highest speed in a blender. The blended samples were separated into two parts, one part was microwaved for 10 s to inactivate the enzymes in the carrots or blueberries, while another part was not microwaved to examine whether and how their inherent enzymes might alter the releasable level of bioactive components. The blended carrot samples were centrifuged at 10,000 g for 5 min, and the supernatant was collected and the calculated volume of acetone was added to make a final concentration of 50% acetone (v/v) for further assays. Blueberries were extracted following the same procedure without cutting. The extracts were kept at 4°C until testing.

### 2.3 Chemical component analysis

The concentrations of β‐carotene in carrot, and malvidin‐3‐*O*‐glucoside and cyanidin‐3‐*O*‐glucoside in blueberry were determined using a Shimadzu LC‐2010 system equipped with a UV detector, binary pump and an auto‐sampler (Shimadzu Technologies, Japan). A luna C‐18 column, 4.6 mm inner diameter × 250 mm and 3.5 μm particle size, was used. HPLC grade water with 0.1% formic acid (v/v) was used as solvent A, and acetonitrile with 0.1% formic acid (v/v) was used as solvent B. The elution was carried out at 5% of solvent B at the beginning, followed by a 10 min linear gradient to 95% B, then back to the initial solvent ratio for next injection. The injection volume was 10 μl, and the oven temperature was 30°C. The identification of target compounds was accomplished by comparing the UV absorption and retention time to that of the standards, and quantification was achieved using UV spectra with standard curve of standard compounds.

### 2.4 Total phenolic contents

The Total phenolic contents (TPC) of the carrot and blueberry samples were measured according to a laboratory procedure described previously (Zhang et al., 2012). Briefly, 100 μl of each 50% acetone extract was mixed with 500 μl of the Folin−Ciocalteu reagent, 1.5 ml of 20% (w/v) sodium carbonate, and 1.5 ml ultrapure water. Absorbance was read at 765 nm after 2 hr of reaction at ambient temperature in the dark. Reactions were conducted in triplicate, and results were reported as milligrams of gallic acid equivalent (GAE) per gram of the fresh sample.

### 2.5 Free radical scavenging properties

#### 2.5.1 Relative DPPH^•^ scavenging capacity

The scavenging capacity against DPPH^•^ was measured following a previously reported method (Niu et al., 2013). Briefly, 100 μl carrot or blueberry extract in 50% acetone, solvent or trolox standard solution was added to 100 μl of freshly prepared DPPH^•^ solution to activate the antioxidant‐radical reaction. The absorbance of each reaction was measured at 515 nm for 40 min. Relative DPPH^•^ scavenging capacity (RDSC) values were calculated based on the areas under the curve relative to trolox standards in the concentration from 7 to 35 μmol/L. Results were expressed as μmol of trolox equivalents (TE) per gram of fresh sample.

#### 2.5.2 Oxygen radical absorbance capacity

The oxygen radical absorbance capacity (ORAC) was evaluated according to a previously reported study (Zhang et al., 2012). Briefly, 225 μl of freshly prepared fluorescein solution was mixed with 30 μl of sample extract, standard or solvent blank in 96‐well plates. The reaction mixtures were incubated at 37°C for 20 min, then added 25 μl of freshly prepared 0.36 mmol/L AAPH to initiate the reactions. The fluorescence of the mixture was measured and recorded using a plate reader once every minute for 2 hr at 37°C, with λ_Ex_ at 485 nm and λ_Em_ at 535 nm. Results were calculated as μmoles of trolox equivalent (TE) per gram of fresh carrot or blueberry.

#### 2.5.3 Hydroxyl radical scavenging capacity

The hydroxyl radical scavenging capacity (HOSC) was estimated following a reported laboratory protocol (Zhang et al., 2012). A total of 170 μl of freshly prepared fluorescein, 30 μl of sample, standard or blank solution, 40 μl of H_2_O_2_ and 60 μl of 3.43 mmol/L FeCl_3_ were mixed. The fluorescence of the mixture was measured and recorded every minute for 2 hr at ambient temperature, with λ_Ex_ at 485 m and λ_Em_ at 535 nm. Results were calculated as μmoles of trolox equivalent (TE) per gram of carrot or blueberry.

### 2.6 Anti‐inflammation effects of carrot and blueberry extracts in RAW 264.7 mouse macrophage cells

RAW 264.7 mouse macrophage cells were cultured in the DMEM containing 10% FBS and 1% amphotericin B/streptomycin/penicillin at 37°C with 5% CO_2_ in air. The RAW 264.7 mouse macrophages were cultured in 6‐well plates overnight to reach a 80% confluence. Each extract was added into cell cultures at 0.2 and 1 mg/ml initial treatment concentrations at 24 hr prior to induction. After 24 hr of pretreatment, LPS was added to the media at an initial concentration of 10 ng/ml. After induction, culture media were discarded, and the cells were collected to perform total RNA isolation and real‐time polymerase chain reaction (PCR).

RNA isolation and real‐time PCR were performed according to previously published protocols (Zhang et al., 2012). After 4 hr of induction with LPS, cells were washed with 1 × PBS, and TRIzol reagent was added for total RNA isolation. IScript advanced cDNA synthesis kit was used to reverse transcribe cDNA. Real‐time PCR was performed on an ABI 7900HT Fast Real‐Time PCR System using AB Power SYBR Green PCR Master Mix. Primers were as follows: IL‐1β (forward, 5′‐ GTTGACGGACCCCAAAAGAT‐3′; reverse, 5′‐CCTCATCCTG‐ GAAGGTCCAC‐3′); IL‐6 (forward, 5′‐CACGGCCTTCCCTACTT‐ CAC‐3′; reverse, 5′‐TGCAAGTGCATCATCGTTGT‐3′); COX‐2 (forward, 5′‐GGGAGTCTGGAACATTGTGAA‐3′; reverse, 5′‐ GCACGTTGATTGTAGGTGGACTGT‐3′). The mRNA amounts were normalized to an internal control, GAPDH mRNA (forward, 5′‐ AGGTGGTCTCCTCTGACTTC‐3′; reverse, 5′‐TACCAGGAAAT‐ GAGCTTGAC‐3′). The following amplification parameters were used for PCR: 50°C for 2 min, 95°C for 10 min, and 46 cycles of amplification at 95°C for 15 s and 60°C for 1 min.

### 2.7 Statistical analysis

Data were reported as the mean ± standard deviation (SD) for triplicate determinations. One‐way analysis of variation (ANOVA) and Tukey's post‐hoc test were employed to identify differences in means. Statistics were analyzed using SPSS for Windows (version release 10.0.5, SPSS, Inc., Chicago, IL). Statistical significance was declared at *p *<* *.05.

## 3 Results and discussion

### 3.1 Chemical component analysis of carrot and blueberry

**Figure 1:**
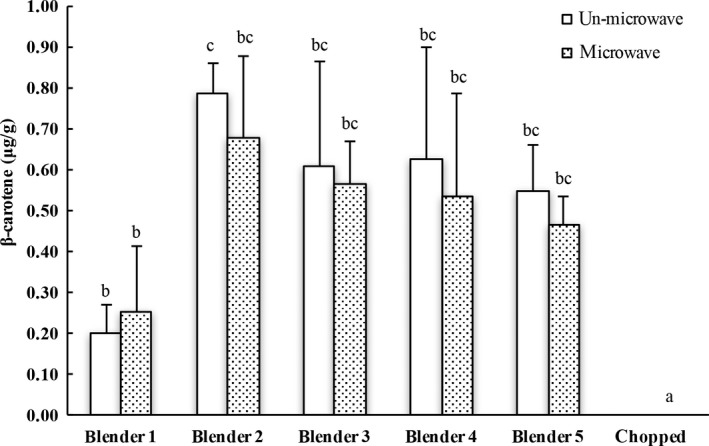
HPLC analysis of β‐carotene in carrot extracts. The results are reported in μg β‐carotene per gram of fresh carrot. The vertical bars represent the standard deviation (*n* = 3) of each data point. Bar with different letter represents significant different at *p* < .05. HPLC, High performance liquid chromatography

β‐carotene is an important carotenoid with vitamin A activities and is rich in carrots. It may quench singlet oxygen and free radicals and reduce the oxidative stress in biological systems, and has been reported for potential health benefits in reducing the risk of cancer, inflammation, coronary heart disease, and other aging‐associated health problems (Jacobo‐Velázquez, Martínez‐Hernández, Del C Rodríguez, Cao, & Cisneros‐Zevallos, 2011). β‐carotene availability from carrots after chopping and grinding with different commercial blenders/grinders was investigated with and without microwave inactivation of the inherent enzymes under the experimental conditions. β‐carotene concentration extracted from the chopped carrot was below the limit of detection (LOD). All tested commercial blenders were able to enhance the release of β‐carotene at a level of 0.2–0.94 μg/g fresh carrot, which was significantly greater than the chopped counterpart (Figure 1). There was no significant difference in extractable concentration of β‐carotene among the blended/ground carrots using different commercial blenders, regardless of microwaving immediately after grinding (Figure 1). In addition, no difference in extractable β‐carotene was observed between the ground carrots using the same blender with and without microwaving. These data suggested that the particle size might alter the release of nutrients or health beneficial components from vegetables such as carrots, although the microwaving could also alter the bioactive release from the matrix.

**Figure 2:**
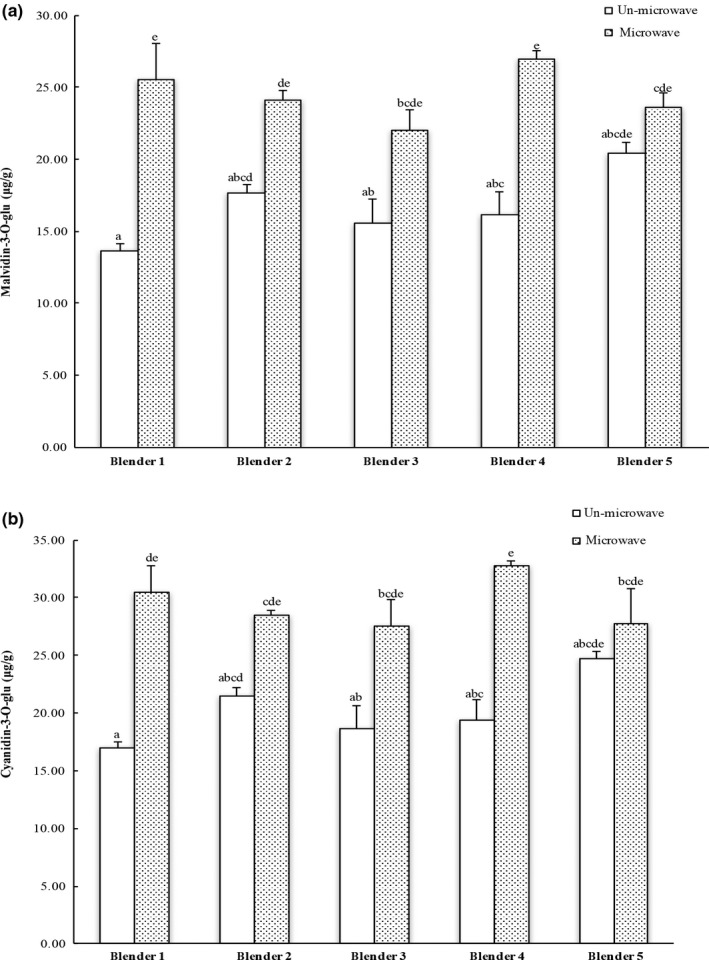
HPLC analysis of (a) malvidin‐3‐*O*‐glucoside and (b) cyanidin‐3‐*O*‐glucoside in blueberry extracts. The results are reported in μg anthocyanins per gram of fresh carrot. The vertical bars represent the standard deviation (*n* = 3) of each data point. Bar with different letter represents significant different at *p* < .05

Anthocyanin is a group of water‐soluble flavonoid glycosides that may appear in red, purple, or blue color. Anthocyanins are well‐recognized natural antioxidants. Dietary anthocyanins may reduce the risk of chronic diseases including cardiovascular diseases (Khanal, Howard, Brownmiller, & Prior, 2009; Kim, Joo, & Yoo, 2009; McGhie, Ainge, Barnett, Cooney, & Jensen, 2003). Malvidin‐3‐*O*‐glucoside and cyanidin‐3‐*O*‐glucoside are two primary anthocyanins in blueberry (Johnson & Gonzalez de Mejia, 2012). The concentration of these two anthocyanins could reflect the total extractable anthocyanins from the blended blueberries. No difference was observed in extractable amount of malvidin‐3‐*O*‐glucoside or cyanidin‐3‐*O*‐glucoside among the blueberries processed using different commercial blenders/grinders regardless (Figure 2a and b, respectively). Interestingly, microwaved blueberries had greater level of extractable malvidin‐3‐*O*‐glucoside or cyanidin‐3‐*O*‐glucoside than its counterpart processed using the same blender/grinder (Figure 2), indicating the potential influence of inherent enzymes such as glucosidases and other carbohydrases (Otieno, Ashton, & Shah, 2007; Alrahmany & Tsopmo, 2012) on bioactive availability from fruits and other botanicals including vegetables. Microwaving immediately after grinding was able to inactivate enzymes, and to prevent the degradation of malvidin‐3‐*O*‐glucoside and cyanidin‐3‐*O*‐glucoside. However, it needs to be pointed out that the reduction in extractable level of malvidin‐3‐*O*‐glucoside or cyanidin‐3‐*O*‐glucoside may not necessarily mean a reduction in the benefits of the extracts to humans, since some degradation products such as malvidin and cyaniding might have greater absorption in GI track. Taking the data in Figure 1 and 2 together, it seemed that both particle size and inherent enzymes might alter the release of beneficial bioactives from carrots and blueberries. Also noted was that the matrix structure or the type of fruits and vegetables may have potential influence on the direction and the level of processing effects on availability of their bioactive components.

### 3.2 TPC and radical scavenging activities of carrot and blueberry

#### 3.2.1 Total phenolic content

**Figure 3:**
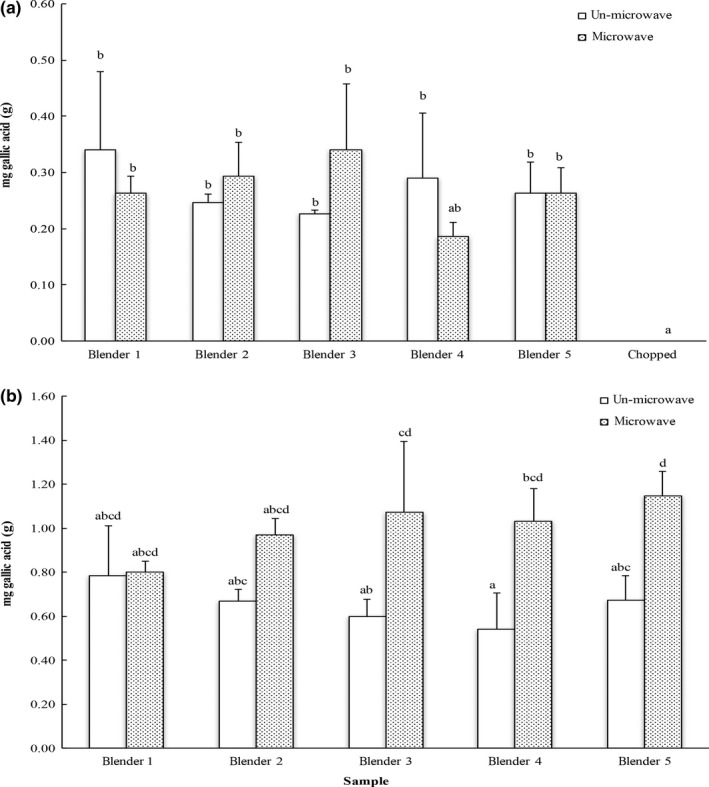
Total phenolic contents (TPC) of (a) carrot and (b) blueberry samples. The results are reported in mg gallic acid equivalents per gram of fresh carrot or blueberry. The vertical bars represent the standard deviation (*n* = 3) of each data point. Bar with different letter represents significant different at *p* < .05

Total phenolic content (TPC) of carrots processed with different blenders with/without microwave is shown in Figure 3a. The TPC values of all the carrot samples were from 0.19 to 0.34 mg gallic acid equivalents per gram of fresh carrot, which was equivalent to 593.6–1062.2 mg chlorogenic acid per kilogram of fresh carrot. This range was greater than that of about 350 mg chlorogenic acid equivalents per kilogram of fresh carrot reported by a previous study (Jacobo‐Velázquez et al., 2011). The difference in TPC values might due to the different carrot varieties, extract method or detect method. Furthermore, no difference in extractable TPC was detected in carrot samples processed with different blenders, and the chopped carrots had detectable TPC and level was lower than that from the blended counterpart under the experimental conditions, suggesting the potential effect of particle size in bioactive release from vegetables. In addition, microwaving had no significant effect on the extractable amount of phenolics from ground carrots

The greatest total phenolic content of blueberry extracts was 0.78 mg gallic acid equivalents per gram of fresh blueberry (GAE/g fresh berry) in the blueberries without microwave treatment after grinding, and that was about 1.13 mg GAE/g blueberry detected in the microwaved blueberry samples (Figure 3b). This TPC value range was 2.44 and 3.53 mg chlorogenic acid equivalents per gram of fresh blueberry, respectively, which was comparable that of 3.35–5.95 mg chlorogenic acid equivalents per gram of fresh blueberry reported before (Connor, Luby, Hancock, Berkheimer, & Hanson, 2002). Different to that observed for carrots, microwaving significantly increased the availability of total phenolic contents in blueberry extracts, indicating that inactivation of blueberry enzymes might reduce the loss of phenolics during grinding and storage of the blended blueberries. Together, the TPC results suggested that microwaving immediately after blending may be recommended for blended blueberry to retain a desirable availability of total phenolics. Again, particle size and inherent enzymes might alter releasable TPC from fruits and vegetables, but the blenders/grinders showed no difference in releasing TPC from either carrots or blueberries.

#### 3.2.2 Radical scavenging capacities of carrot and blueberry

Radical scavenging agents are well recognized for their potential health beneficial effects. In general, two or more radical systems are needed to investigate the scavenging abilities of a selected antioxidant since radical system may alter the results of antioxidant property assessment (Niu et al., 2013). The present study evaluated whether and how carrots and blueberries processed using different blenders and chopping with and without microwaving might alter the available scavenging components or activities against DPPH, OH, and oxygen radicals.

**Figure 4:**
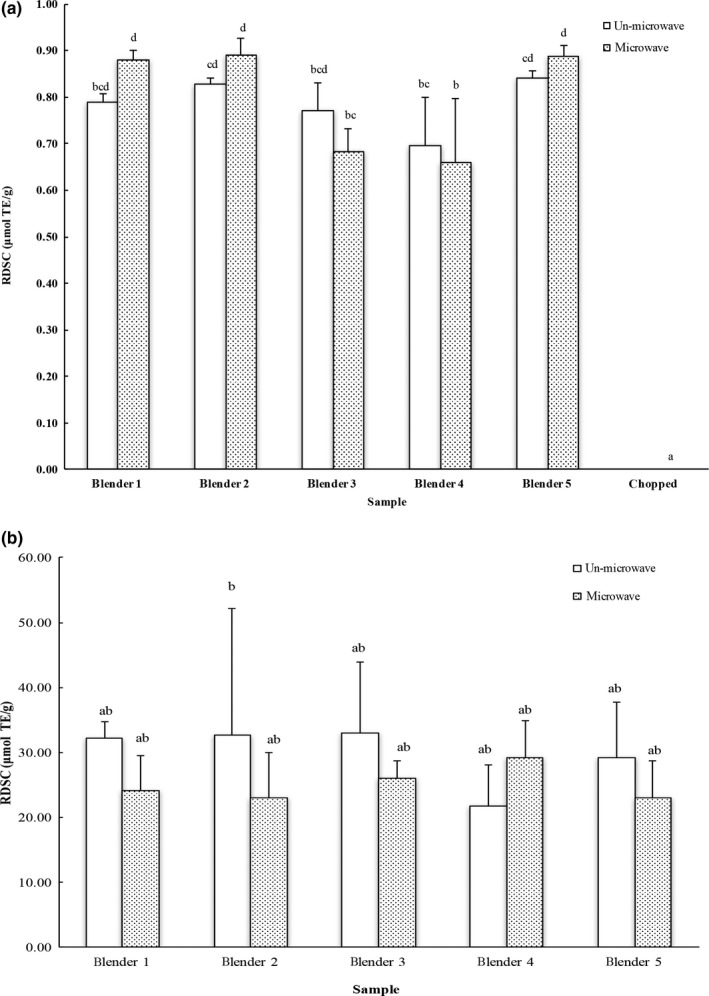
Relative DPPH scavenging capacity (RDSC) of (a) carrot and (b) blueberry samples. The results are reported in trolox equivalents per gram of fresh weight. The vertical bars represent the standard deviation (*n* = 3) of each data point. Bar with different letter represents significant different at *p* < .05

DPPH^•^ scavenging capacities of carrot samples processed using home‐use blenders without/with microwave were 0.70–0.84 and 0.66–0.89 μmol trolox equivalent per gram of fresh carrot, respectively. These values are significantly greater than that of the chopped carrots with nondetectable DPPH^•^ scavenging capacity (Figure 4a). Microwaving did not alter the DPPH^•^ scavenging capacity of carrots. Previous studies reported 0.18 mg trolox equivalents per mL of carrot oil (Shebaby et al., 2013), which is about 0.025 μmol trolox equivalent per gram of carrot seeds with a 3.47% oil.

DPPH radical scavenging capacities of the ground blueberry samples with different blenders without/with microwave were 21.70–32.99 and 22.95–29.19 μmol trolox equivalent per gram of fresh blueberry, respectively. No difference was observed between the tested blenders in their ability to promote the antioxidant release from blueberries (Figure 4b). Furthermore, microwaving had no influence on available DPPH radical scavenging capacities of the ground blueberries.

#### 3.2.3 ORAC of carrot and blueberry

**Figure 5:**
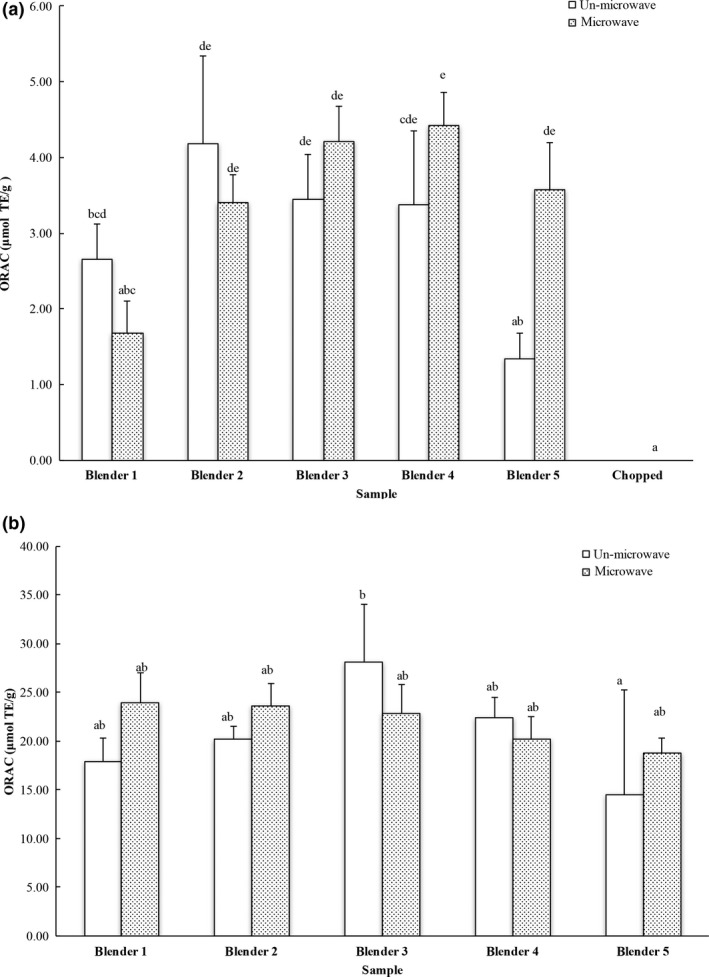
Oxygen radical absorbing capacity of (a) carrot and (b) blueberry samples. The results are reported in trolox equivalents per gram of fresh weight. The vertical bars represent the standard deviation (*n* = 3) of each data point. Bar with different letter represents significant different at *p* < .05

The Oxygen radical absorbance capacity (ORAC) values of the ground carrots prepared with different blenders/grinders without/with microwave were 1.34–4.19 and 1.68–4.42 μmol trolox equivalent per gram of fresh carrot, respectively (Figure 5a). This was comparable to that of 1.48 and 5.39 μmol trolox equivalents per gram of fresh carrot from a previous study (Seljåsen et al., 2013). The chopped carrot samples showed the lowest ORAC values compared to the blended or ground ones, indicating the importance of particle size on antioxidant release from carrots. Furthermore, microwaving was able to induce a significant increase of ORAC in one of the five ground carrots using a commercial blender, suggesting a possible effect from the inherent carrot enzymes.

The ORAC values of blueberry extracts prepared with different blenders without/with microwave were 14.47–28.14 and 18.73–23.95 μmol trolox equivalents per gram of fresh blueberry, respectively. This range was comparable to that of 4.6–30.5 μmol trolox equivalents per gram of fresh blueberry with the average value of 15.9 μmol trolox equivalents per gram of fresh blueberry (Ehlenfeldt & Prior, 2001). No difference was observed among blueberry samples processed using different blenders, regardless of microwave inactivation of inherent enzymes (Figure 5b).

#### 3.2.4 HOSC of carrot and blueberry

**Figure 6:**
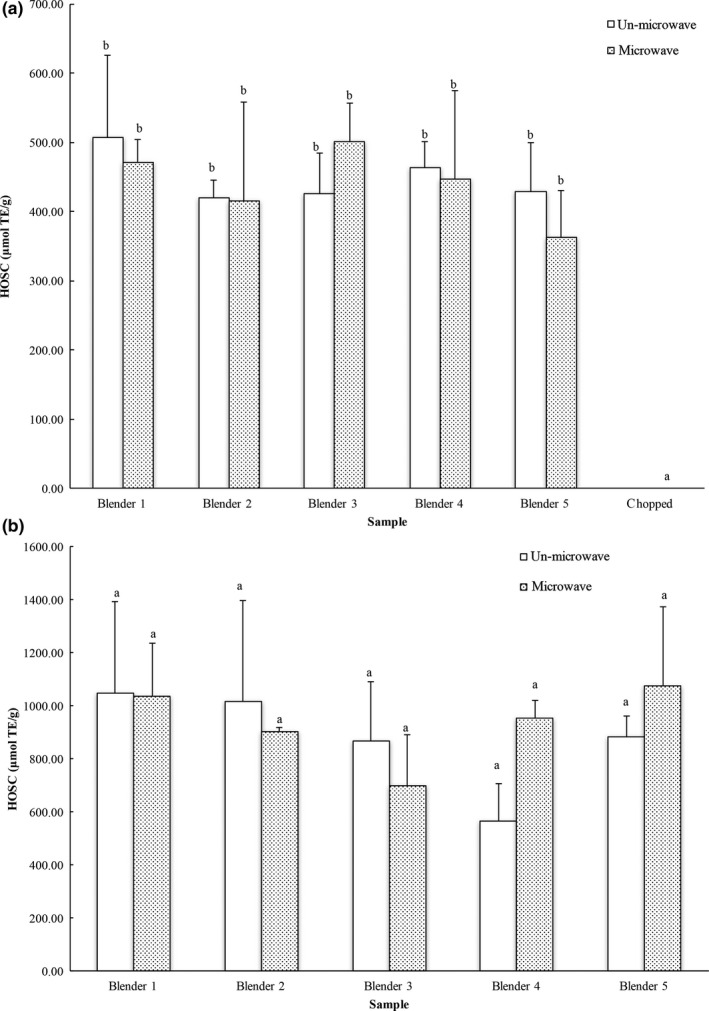
Hydroxyl radical scavenging capacity of (a) carrot and (b) blueberry samples calculated to trolox equivalents (TE) on a per fresh weight basis. The vertical bars represent the standard deviation (*n* = 3) of each data point. Bar with different letter represents significant different at *p* < .05

Hydroxyl radical is one of the most reactive radical species generated in biological systems that would be associated with oxidative damage at cellular level and lead to the development of numerous chronic diseases. The tested blenders had no difference in their ability to promote the release of Hydroxyl radical scavenging capacity (HOSC) from carrots, and the carrots ground with the blenders had significantly greater extractable HO^•^ scavenging components (Figure 6a). The HOSC values of carrot extracts prepared with different blenders without/with microwave were 425.80–506.98 and 362.70–500.96 μmol trolox equivalents/g fresh carrot for the blended carrots, respectively.

No difference between blenders was observed in their ability to enhance the availability of HO^•^ scavenging components from blueberries (Figure 6b). The HOSC values of the blueberry samples prepared with different blenders without/with microwave were 565.86–1047.53 and 698.50–1077.79 μmol trolox equivalents/g fresh weight under the experimental conditions, respectively. In addition, microwaving had no significant influence on the extractable levels of HO^•^ scavenging components from carrots and blueberries.

### 3.3 Anti‐inflammatory capacities of carrot and blueberry

**Figure 7:**
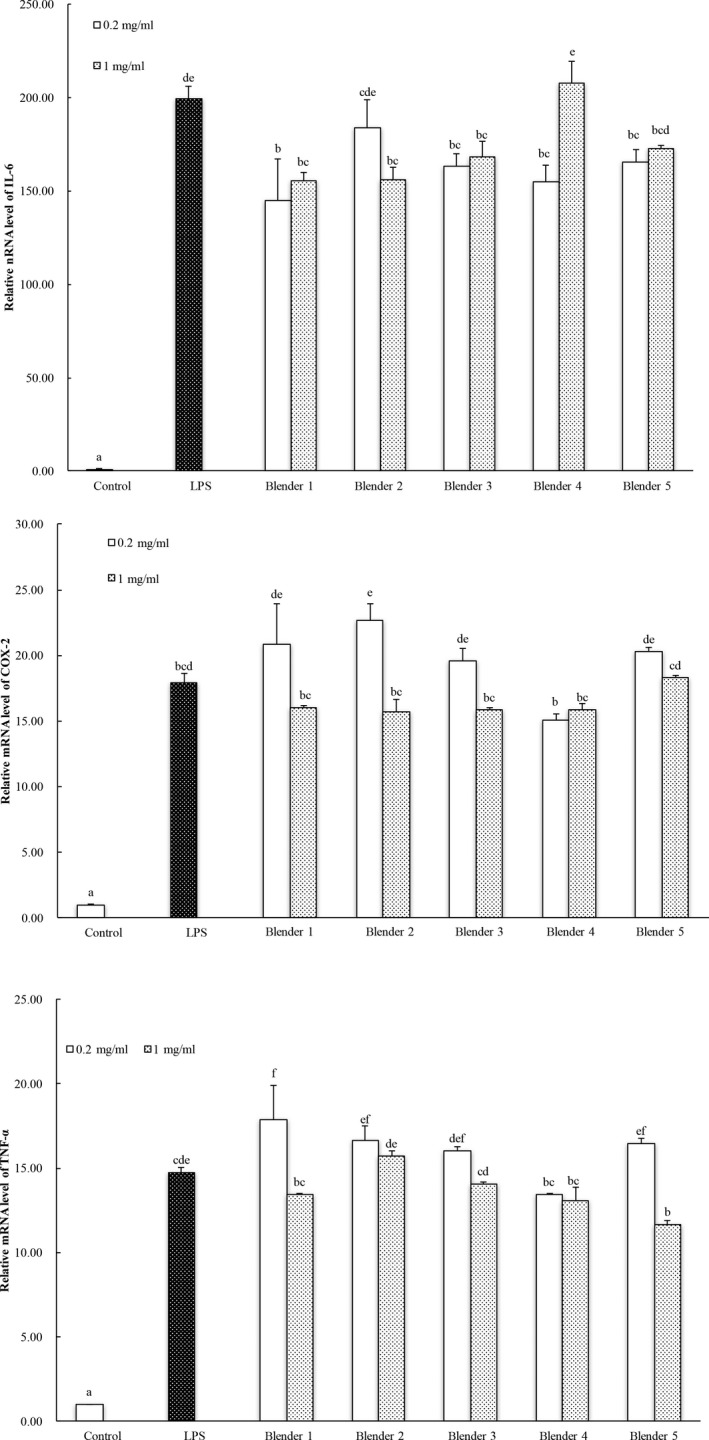
Anti‐inflammatory effects of carrot extracts in RAW 264.7 mouse macrophage cells. Control and LPS contained the same concentration of DMSO as all the treatment samples. LPS stands for lipopolysaccharide. The vertical bars represent the standard deviation (*n* = 3) of each data point. Bar with different letter represents significant different at *p* < .05

Upon LPS (lipopolysaccharide) stimulation of macrophage, nuclear factor‐κB pathway would be activated and result in a release of variety of inflammatory factors including tumor necrosis factor‐α (TNF‐α) and interleukin 6 (IL‐6). Other pathways such as COX‐2 (cyclooxygenase 2) regulated pathway could also be triggered during inflammation. The potential anti‐inflammatory effects of carrot extracts prepared using the selected commercial blenders/grinders are reported in Figure 7. No difference was observed in the carrots samples prepared using different commercial blenders/grinders in releasable levels of potential anti‐inflammatory components in the LPS‐induced IL‐6 mRNA expression in macrophage cells (Figure 7). The inhibitory effects of blueberry extracts on LPS‐induced COX‐2 and TNF‐α mRNA expressions could be dose‐dependent.

**Figure 8:**
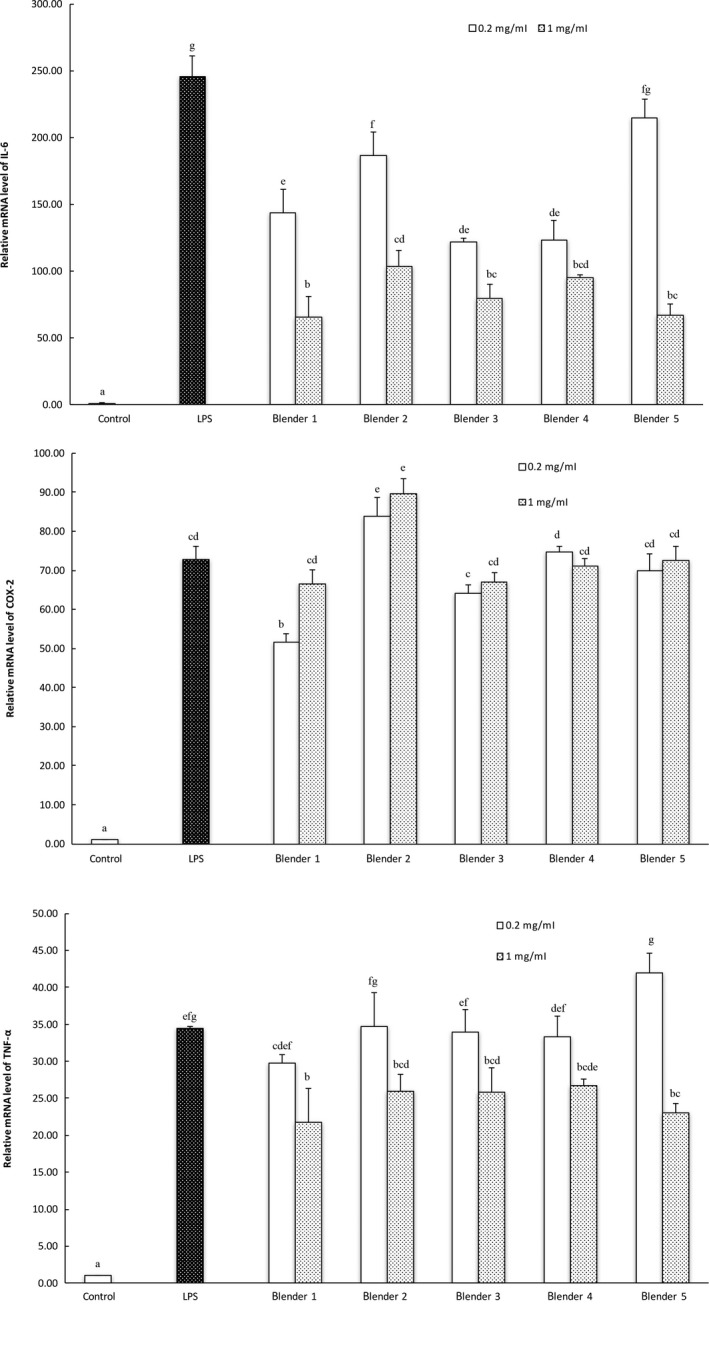
Anti‐inflammatory effects of blueberry extracts treated with RAW 264.7 mouse macrophage cells. Control and LPS contained the same concentration of DMSO as all the treatment samples. LPS stands for lipopolysaccharide. The vertical bars represent the standard deviation (*n* = 3) of each data point. Bar with different letter represents significant different at *p* < .05

Blueberry extracts prepared using different commercial blenders were able to dose‐dependently reduce the LPS –induced IL‐6 and TNF‐α mRNA expressions in the cultured macrophages (Figure 8), but not COX‐2 mRNA expression (Figure 8b). No difference was observed in the blueberry samples prepared using different commercial blender/grinders in releasable levels of potential anti‐inflammatory components in the LPS‐induced macrophage cells (Figure 8).

In conclusion, the present study tested and compared selected commercial blenders and grinders for their effects on releasing health beneficial components from fruits and vegetables using carrot and blueberry as probe foods. The results indicated no difference among the commercial blenders/grinders on the extractable levels of health beneficial components including carotenoids, anthocyanins, free radical scavenging compounds and potential anti‐inflammatory components, except for the blender 1 in releasing significantly lower amount of β‐carotene from carrot. In another words, processing or preparation using an expensive blender or grinder is not always associated with a greater level of beneficial bioactives in the processed foods such as a fruit or vegetable smooth. The results from this study also suggested that food preparation or processing procedure might alter the overall amount of available bioactives. The findings are important for consumers in selecting their home food preparation approaches to obtain desirable health.

## Conflict of Interest

None declared.
